# The Role for Dickkopf-Homolog-1 in the Pathogenesis of Crohn’s Disease-Associated Fistulae

**DOI:** 10.1371/journal.pone.0078882

**Published:** 2013-11-08

**Authors:** Sandra Michaela Frei, Colette Hemsley, Theresa Pesch, Silvia Lang, Achim Weber, Ekkehard Jehle, Anne Rühl, Michael Fried, Gerhard Rogler, Michael Scharl

**Affiliations:** 1 Division of Gastroenterology and Hepatology, University Hospital Zurich, Zurich, Switzerland; 2 Department of Pathology, Institute of Surgical Pathology, University Hospital of Zurich, Zurich, Switzerland; 3 Department of Surgery, Oberschwaben-Klinik, Ravensburg, Germany; 4 Private Practice, Lörrach, Germany; 5 Zurich Center for Integrative Human Physiology, University of Zurich, Zurich, Switzerland; Rush University Medical Center, United States of America

## Abstract

**Background:**

One of the most challenging conditions in Crohn’s disease (CD) patients is the treatment of perianal fistulae. We have recently shown that epithelial-to-mesenchymal transition (EMT) plays a crucial role during CD-fistulae development. Dickkopf-homolog 1 (DKK-1) is known to play a key role during EMT. Here, we investigated a role for DKK-1 in the pathogenesis of CD-associated fistulae.

**Methods:**

Dkk-1 protein expression in CD-fistula specimens were investigated by immunohistochemistry. Colonic lamina propria fibroblasts (CLPF) were obtained from either non-IBD control patients or patients with fistulizing CD. HT-29 intestinal epithelial cells (IEC) were either grown as monolayers or spheroids. Cells were treated with either TNF-α, TGF-β or IL-13. Knock-down of DKK-1 or β-Catenin was induced in HT-29-IEC by siRNA technique. mRNA expression was determined by real-time-PCR.

**Results:**

Dkk-1 protein was specifically expressed in transitional cells lining the fistula tracts. TGF-β induced DKK-1 mRNA expression in HT-29-IEC, but decreased it in fistula CLPF. On a functional level, DKK-1 knock-down prevented TGF-β-induced IL-13 mRNA expression in HT-29-IEC. Further, loss of β-Catenin was accompanied by reduced levels of DKK-1 and, again, IL-13 in IEC in response to TGF-β. In turn, treatment of HT-29-IEC as well as fistula CLPF with IL-13 resulted in decreased levels of DKK-1 mRNA. Treatment with TNF-α or the bacterial wall component, muramyl-dipeptide, decreased DKK-1 mRNA levels in HT-29-IEC, but enhanced it in fistula CLPF.

**Discussion:**

We demonstrate that DKK-1 is strongly expressed in cells lining the CD-fistula tracts and regulates factors involved in EMT initiation. These data provide evidence for a role of DKK-1 in the pathogenesis of CD-associated perianal fistulae.

## Introduction

Perianal and intestinal fistulae represent a frequent complication in up to 50% of patients with Crohn’s disease (CD) [1]–[3], one of the major types of inflammatory bowel diseases (IBD), and pose a challenging problem, since current treatment options such as administration of antibiotics [4], immunosuppressants or tumor necrosis factor alpha (TNF-α) antibodies often are insufficient to cure the complication and achieve complete fistula closure [5]–[8]. Surgical intervention is frequently required, however, often not providing a definitive cure.

So far, the pathogenesis of fistula formation in CD is poorly understood. A better knowledge is essential to improve and develop targeted therapeutic strategies. In morphological studies, we have recently demonstrated that some remaining epithelial cells are present on the inner surface of fistula tracts while other cells show mesenchymal or myofibroblast-like features. The latter have been categorized as so-called “transitional cells” (TC) [9]. Our previous data demonstrate that in CD fistulae the polarized epithelial cells are undergoing transformation into TC, a process known as epithelial-mesenchymal transition (EMT) [10]–[11]. These TCs express both, mesenchymal markers, like increased levels of vimentin and α-SMA, but coincidentally also typical epithelial cell markers such as cytokeratine-8 and -20 [11]–[14].

EMT plays a crucial role in physiological processes such as embryogenesis, organ development, wound repair and tissue remodeling, but is also involved in pathological processes like tissue fibrosis or cancer progression [15]–[17]. The most powerful inducer of EMT is tumor growth factor beta (TGF-β) [18]–[19]. TGF-β causes the epithelial cells to lose polarity and cell-cell interactions, characterized by down-regulation of E-Cadherin mediated by its repressor Snail1 [20]–[21]. Additionally, TGF-β induces β-Catenin translocation into the nucleus to regulate target gene expression. Notably, TGF-β can be induced by TNF-α [22]. Although TGF-β is the most powerful EMT inducer, Bates et al. showed that also TNF-α is able to induce EMT in colonic organoids [23].

Recently we demonstrated that TC up-regulate expression of genes associated with cell migration and invasion, such as SNAIL1, SLUG (SNAIL2) and β6-integrin [11], [12]. Further, we detected strong expression of interleukin-13 (IL-13) in TC lining the tracts of CD-associated perianal fistulae and revealed that IL-13 secretion can be induced by TGF-β in fibroblasts derived from fistulizing CD-patients. IL-13 then causes an increased expression of β6-integrin and the transcription factor SLUG in HT-29 cells [12]. We also found that SNAIL1 levels in TGF-β- as well as SLUG and β6-integrin levels in IL-13-exposed IEC are clearly increased, providing a further hint to the onset of EMT [12].

An important factor involved in the regulation of cell migration is Dickkopf-homolog-1 (DKK-1). Loss of DKK-1 has been associated with progression of certain types of cancers, such as colorectal carcinoma (CRC) [24]–[26], [28]. On a functional level, the secreted glycoprotein is capable to block IEC migration [27]–[28]. Additionally, DKK-1 is a potent antagonist of the canonical Wnt/β-Catenin signaling pathway and has been implicated to act as a mediator of inflammation [27]. Wnt pathway regulates Dkk-1 expression through β-Catenin/T-cell factor (TCF), in particular TCF-4 [28].

Here, we show that TNF-α, TGF-β and IL-13 are involved in regulatory feedback loops likely controlling cell migration and invasion in the pathogenesis of CD-associated perianal fistulae. Unfortunately, since there is no well-established animal model available for studying fistula development in CD, our data are limited to the presented *ex vivo* and *in vitro* approaches. Nevertheless, our data demonstrate for the first time that TGF-β-stimulated IL-13 secretion might be regulated via DKK-1, strongly suggesting an involvement of DKK-1 in the pathogenesis of CD-associated perianal fistulae.

## Materials and Methods

### Cell Lines

HT-29-IECs of human origin were either grown as monolayers or spheroids in a humidified atmosphere with 10% CO_2_ at 37°C in low glucose Dulbecco’s modified Eagle’s medium (Invitrogen, Carlsbad, CA) containing 1000 mg/L D-glucose, L-glutamine and sodium pyruvate which was additionally supplemented with 1% penicillin/streptomycin solution (Invitrogen) and 5% fetal calf serum (FCS; VWR).

### Patient Samples

Both genders were included in our studies. Tissue samples from healthy control subjects, patients with active ulcerative colitis (n = 3), ulcerative colitis in remission (n = 3), active Crohn’s disease (n = 5), Crohn’s disease in remission (n = 4) and perianal fistula specimens from CD-patients (n = 8) were used for immunohistochemical stainings. These specimens were surgically resected, transferred into 4% formalin and stored at 4°C until further analyses were performed. Intestinal fibroblasts of non-IBD-subjects and fistulizing CD-patients were cultivated using mentioned surgical specimens. The procedure of fibroblast isolation is described below.

Written informed consent was obtained and the studies were approved by the Cantonal Ethics Committee Zürich (Approval no. EK-1755) before specimens were collected. All investigations have been conducted according to the principles expressed in the Declaration of Helsinki.

### Isolation and Culture of Human Colonic Lamina Propria Fibroblasts (CLPF)

Primary CLPF cultures were obtained from seven fistulizing CD-patients (mean age 53±5 yrs). Fistula tracts of surgical specimens were initially cut into pieces of 1 mm thickness and rinsed in fibroblast medium consisting of high glucose Dulbecco’s modified Eagle’s medium (DMEM; PAA, Cölbe, Germany) supplemented with 10% FCS (Gibco, Karlsruhe, Germany) and cultured in 25 cm^2^ culture flasks (Costar, Bodenheim, Germany) with DMEM containing 10% FCS, penicillin (100 IU/ml), streptomycin (100 µg/ml), ciprofloxacin (8 µg/ml; Bayer), gentamycin (50 µg/ml; Invitrogen) and amphotericin B (1 µg/ml; Roth). The tissue was washed and digested for 30 min at 37°C in phosphate buffered saline (PBS, Gibco) containing calcium and magnesium ions (PAA), 1 mg/ml collagenase I (Sigma, St. Louis, MO), 0.3 mg/ml DNase I (Boehringer, Mannheim, Germany) and 2 mg/ml hyaluronidase (Sigma). Thereafter, the isolated cells were rinsed again. The tissue pieces from the fistula were put onto scratched petri dishes containing fibroblast medium until fibroblast migration was reached. Cells were further grown in a humidified atmosphere with 10% CO_2_ at 37°C in high glucose Dulbecco’s modified Eagle’s medium containing 10% FCS, an antibiotic agent (alternately ciprofloxacin, 8 µg/ml or gentamycin, 50 µg/ml), 1% non-essential amino acid solution (NEAA), 1% sodium pyruvate (Sigma-Aldrich), 1% vitamin solution and 1% L-glutamine (Sigma).

The same procedure was performed for isolation of intestinal control fibroblasts deriving from six non-IBD subjects (mean age 49±12 yrs).

### Immunohistochemistry

Immunohistochemical studies were performed using a peroxide based method on tissue specimens fixed in formalin and embedded in paraffin. The tissue samples were first deparaffinized with Histoclear® (Brunschwig), descending concentrations of ethanol, and rehydrated. Antigen retrieval was performed using citrate buffer, pH 6.0 (DAKO, Glostrup, Denmark) for 45 min at 98°C in a water bath and cooled down to room temperature (RT). Thereafter, tissues were washed in PBS. Endogenous peroxidases were eliminated by incubation with 0.9% hydrogen peroxide for 15 min at RT. The specimens were washed in PBS and blocked with 1% BSA in PBS in a wet chamber for 1 h at RT, mouse-anti-DKK-1-antibody (1 mg/ml; Abnova) added, incubated for 3 h at RT and rinsed with PBS. Secondary antibody (EnVision™ System anti-mouse-HRP-labeled polymer from DAKO) was applied for 3 h at RT. To visualize the antibody binding, DAB method was used according to manufacturer’s instructions (DAKO). The samples were counterstained with hematoxyline and dehydrated in ascending concentrations of ethanol and Histoclear® and finally mounted in Pertex™. Microscopic assessment were performed using an AxioCam MRc5 (Zeiss, Jena, Germany) on a Zeiss Axiophot microscope (Zeiss) with Axio Vision Release 4.7.2 software (Zeiss).

### Western Blotting

HT-29 whole cell lysates obtained using mammalian protein extraction reagent (MPER; Thermo Scientific) were mixed with an equal amount of 2× gel loading buffer (50 mmol/L Tris, pH 6.8, 2% sodium dodecyl sulfate, 200 mmol/L dithiothreitol, 40% glycerol, 0.2% bromophenol blue) and heated up to 95°C for 10 minutes. By sodium dodecyl sulfate–polyacrylamide gel electrophoresis, proteins were separated and transferred onto nitrocellulose membranes (Brunschwig). Membranes were blocked with a blocking solution containing 3% milk and 1% BSA. The membranes were incubated over night at 4°C with an anti-Dkk-1 monoclonal antibody (Novus Biologicals; dilution of 1∶1000), an anti-β-Catenin polyclonal antibody (Cell Signaling; dilution of 1∶1000; produced in rabbit) or an anti-β-Actin monoclonal antibody (Sigma; dilution of 1∶3000, produced in mouse). Membranes were washed 3 times with tris-buffered saline containing 0.1% tween 20 (0.1% TBST) for 10 minutes, horseradish-peroxidase–labeled secondary anti-mouse- or anti-rabbit–IgG-antibody (Santa Cruz Biotechnology) in the mentioned blocking solution was added for 1 hour at RT and membranes were washed 3 times for 10 minutes with TBST. Proteins were detected using an enhanced chemiluminescence detection kit (GE Healthcare).

### Stimulation of HT-29-IEC

HT-29 were re-suspended in 500 µl HT-29 medium and seeded onto inserts in 24 well plates. 500 µl HT-29 medium was added outside the inserts. Cells were grown for five days, medium removed and replaced by low glucose DMEM without additives. After starvation, HT-29 cells were basolaterally stimulated with either 100 ng/ml human recombinant Interleukin-13 (rhIL-13, R&D), 100 µg/ml of an anti-IL-13 antibody (provided by Novartis AG, Basel, Switzerland), 50 ng/ml human recombinant tumor necrosis factor alpha (TNF-α, Calbiochem/Merck, VWR), 50 ng/ml human recombinant tumor growth factor beta (TGF-β1, Calbiochem/Merck, VWR), 100 ng/ml muramyl dipeptide (MDP, InvivoGen) in a dimethyl sulfoxide (DMSO) – FuGene transfection reagent (Promega) solution, 100 ng/ml ultra-pure lipopolysaccharide (LPS, from E. coli, InvivoGen) and/or 24 µg/ml of an anti-TNF-antibody. The concentrations used for IL-13, anti-IL-13 antibody, TNF-α and TGF-β for the stimulation of the cells were adopted from Scharl M. et al. [12].

### Stimulation of Human CLPF

Primary fibroblasts re-suspended in 2 ml fibroblast medium of healthy control and fistulizing CD-patients were seeded into six well plates and grown until high grade confluence. Medium was replaced by DMEM without additives. Stimulation was performed as described above.

### Spheroids as *in-vitro* Model for EMT

HT-29-IECs were used for cultivation of spheroids as three-dimensional cell culture model for EMT [12]. 4.5×10^3^ cells per spheroid were re-suspended in 24 µl HT-29 medium. Spheroids were then seeded in Terasaki plates (GREINER–Microtest-Plates, TC; EBIS/Huber & Co) with 60 cavities and grown for seven days. At day seven after seeding, spheroids were stimulated with either 1 µl HT-29 medium, 50 ng/ml TGF-β or 100 ng/ml IL-13 in 1 µl HT-29 medium. Spheroids were harvested at different time points.

### Real-time Polymerase Chain Reaction (RT-PCR)

Cells were lyzed in RLT-buffer (Qiagen, Valencia, CA) containing 1% β-mercaptoethanol. RNA was isolated using RNeasy Plus Mini Kit (Qiagen, Valencia, CA) using a QIA-Cube (Qiagen) and DNA was removed by DNase I (Qiagen) according to manufacturer’s instructions. RNA concentration was determined by absorbance at 230 nm using NanoDrop ND1000 (Thermo Scientific). cDNA synthesis was performed using a High Capacity cDNA Reverse Transcription Kit (Applied Biosystems, Foster City, CA). TaqMan Assays and TaqMan Gene Expression Master Mix were obtained from Applied Biosystems. RT–PCR was performed in a 7900HT Fast RT–PCR system using SDS 2.2 software (Applied Biosystems). Conditions were set on 45 cycles, an enzyme activation of 20 s at 95°C, 1 s denaturation at 95°C and the anneal/extend at 60°C for again 20 s. Triplicate measurements were performed. Results were analyzed with ▵▵CT-method. Measured values were normalized to endogenously expressed β-Actin.

### Small Interfering RNA (siRNA) Transfection

A transient knock-down of β-Catenin or DKK-1 was reached using siRNA constructs specific either for β-Catenin or DKK-1 (both from Applied Biosystems) in HT-29-IEC. 1×10^6^ IEC were re-suspended with 100 µl Mirus® Ingenio™ Electroporation Solution (Mirus Bio LLC, Madison, WI) either without specific siRNA constructs, serving as control, or with 5 nmol standard purity of three different annealed β-Catenin- or DKK-1-specific Silencer® Pre-designed siRNA oligonucleotides. Cell transfection was performed using the Amaxa nucleofector system (Lonza, Walkersville, MD). Transfected cells were cultured on filter membranes for two days before they were stimulated with either TNF-α, TGF-β or IL-13. Medium was changed 24 h after transfection to remove the remaining electroporation reagent.

### Statistical Analysis

Data are shown as means +/− S.D. for a series of n experiments. Statistical analyses were performed with InStat3™ software (GraphPad software Inc.) by one way ANOVA followed by Student-Newman-Keuls post hoc test. P-values <0.05 were considered significant.

## Results

### Dkk-1 is Expressed along Fistula Tracts in CD

**Figure 1:**
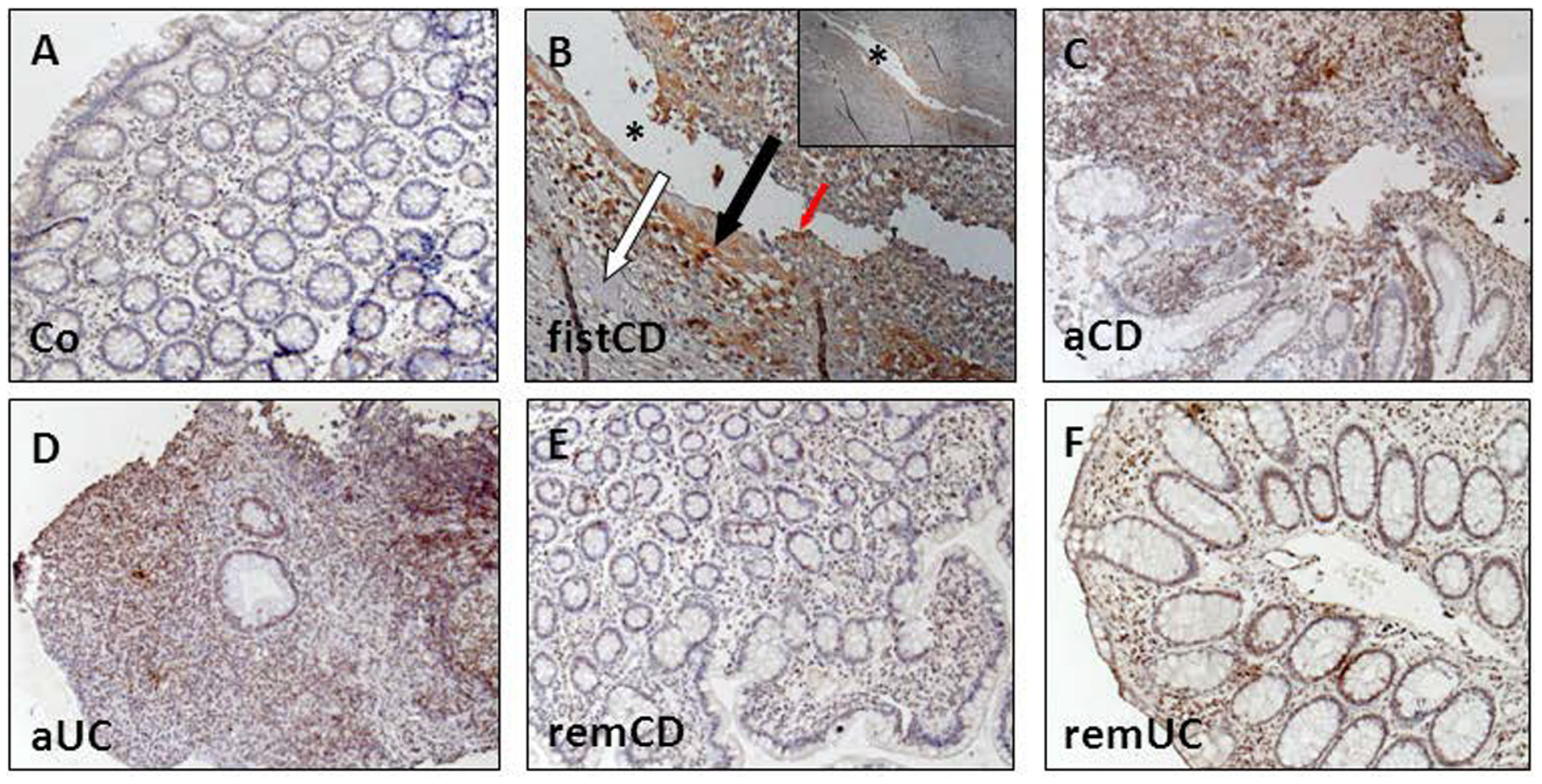
Dkk-1 is strongly expressed in TCs lining the fistula tract of CD patients. Immunohistochemical stainings of healthy control subjects (Co, A), fistula tissue derived from CD-patients (fistCD, B), CD-patients with active disease (aCD, C), patients with an active ulcerative colitis (aUC, D), CD patients in remission (remCD, E) and colitis patients in remission (remUC, F) were analyzed. The representative section of the fistula associated to CD highlights the presence of Dkk-1 in TCs lining the fistula tract (red arrow), cells covering the fistula tract (black arrows), whereas surrounding cells show almost no staining signal (white arrows). Asterisks indicate the fistula tract. Pictures are acquired with a 10-fold magnification.

Dkk-1 has been associated with an altered migratory potential of epithelial cells. Since we recently found that genes involved in cell migration and invasion are expressed along or around the tracts of CD-associated perianal fistulae [11]–[12], we assessed whether Dkk-1 would be present in fistula specimens of CD-patients. In immunohistochemically stained intestinal tissue samples derived from non-IBD control patients, Dkk-1 staining was very weak (Figure 1, A). In tissue samples from CD-patients with a perianal fistula (n = 9) Dkk-1 staining was detectable in TCs lining the fistula tracts (Figure 1, B, red arrow). Dkk-1 also was detected in the fibrotic areas adjacent to the fistula tracts (Figure 1, B, black arrow), while fistula surrounding areas consisting of inflammatory cells almost were free of Dkk-1 staining (Figure 1, B, white arrow).

In acute inflamed intestinal tissue during active CD (Figure 1, C) and UC (Figure 1, D), we found a strong staining signal for Dkk-1, representing a high cell turnover and, likely, ongoing wound repair mechanisms. In contrast, Dkk-1 staining was weak in IEC of regular colonic crypts in patients with CD (Figure 1, E) and UC (Figure 1, F) in remission comparable to healthy control tissue (Figure 1, A). In the positive control, human uterus, strong Dkk-1 staining was detectable (Figures S1, A–B), whereas the negative control revealed no unspecific staining in a perianal fistula (Figures S1, C–D).

On mRNA level, we detected increased levels of DKK-1 mRNA in the respective intestinal tissue samples from patients with active CD and UC when compared to samples from non-IBD control patients or patients with CD in remission fully supporting our observations by IHC (data not shown). These data demonstrate that Dkk-1 protein is highly expressed in TCs lining the tracts of CD-associated fistulae and suggest that Dkk-1 might be relevant during fistula pathogenesis.

### TGF-β Induces DKK-1 mRNA Expression in IEC, but Decreases it in CD Fistula CLPF

**Figure 2:**
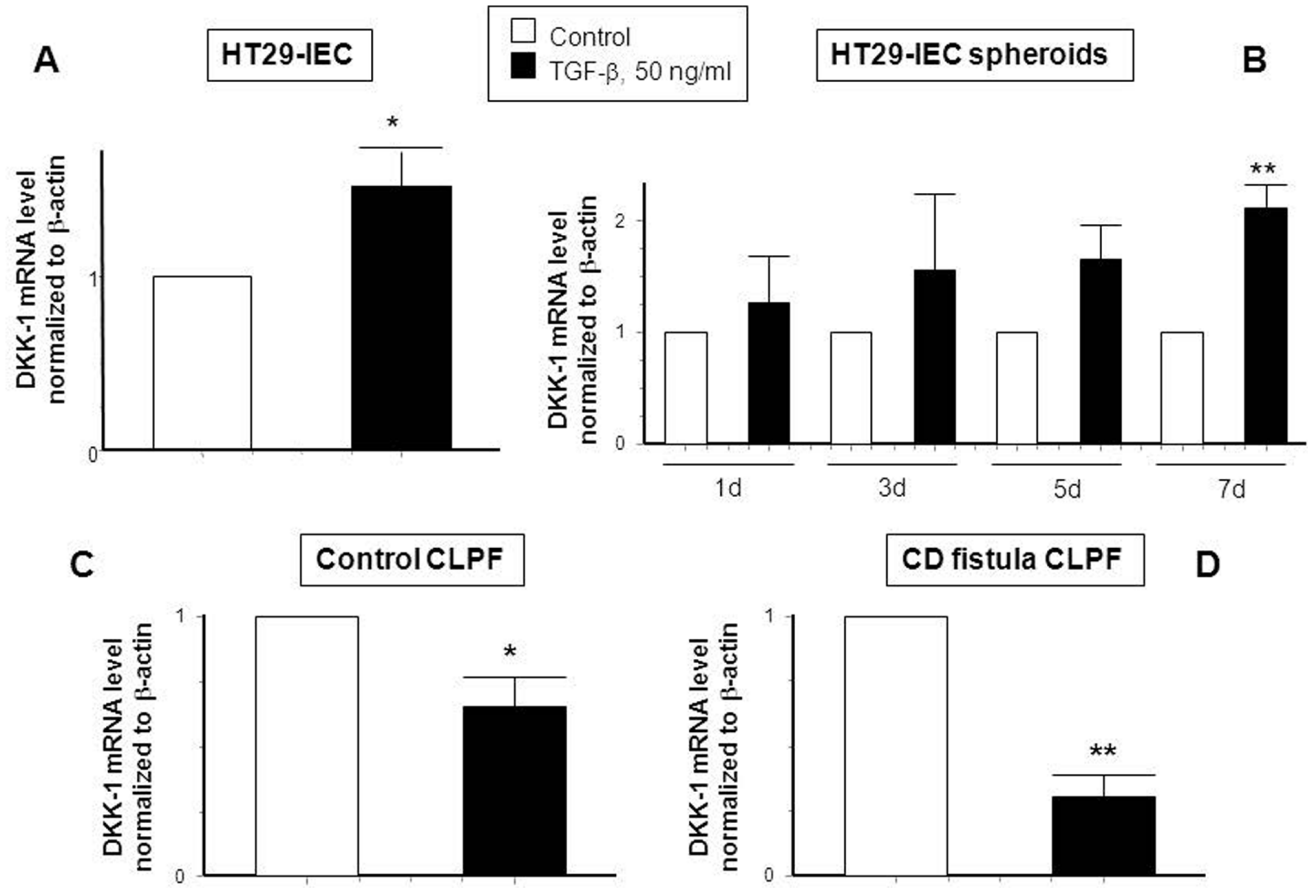
TGF-β induces mRNA expression of DKK-1 in human IEC, but reduces it in control and CD-derived fistula fibroblasts. DKK-1 mRNA expression is demonstrated in (A) HT-29 cells treated with TGF-β (50 ng/ml) for 24 h (n = 3), (B) HT-29 spheroids treated with TGF-β for 1, 3, 5 or 7 days, fibroblasts of (C) non-IBD-control patients (n = 3) and (D) CD-associated fistulae (n = 3), both treated with TGF-β for 24 h. Asterisks indicate significant differences versus the respective control. * = p<0.05, ** = p<0.01.

TGF-β is the most important trigger of EMT [18]–[19], [22]. Therefore, we assessed the effect of TGF-β on DKK-1 expression in IEC and CLPF. Application of 50 ng/ml TGF-β significantly increased DKK-1 mRNA expression in HT-29 monolayers after treatment for 24 h (Figure 2, A). Of note, protein levels of Dkk-1 were also increased after a treatment of HT-29 cells treated with TGF-β for 24 h (Figure S2, A). Correspondingly, in the HT-29-spheroid cell EMT-model DKK-1 mRNA was already increased upon TGF-β stimulation after 1 day and this effect reached statistical significance after treatment for 7 days (Figure 2, B). In contrast, in both, control and CD CLPF, TGF-β significantly down-regulated DKK-1 mRNA expression by treatment for 24 h (Figure 2, C–D). These findings demonstrate that TGF-β exerts different effects on DKK-1 expression in IEC and CLPF. While the growth factor increases DKK-1 mRNA in IEC, it reduces DKK-1 mRNA in CLPF derived from healthy non-IBD-subjects and CD-patients with a fistulizing disease course.

### TGF-β Induces IL-13 via DKK-1

**Figure 3:**
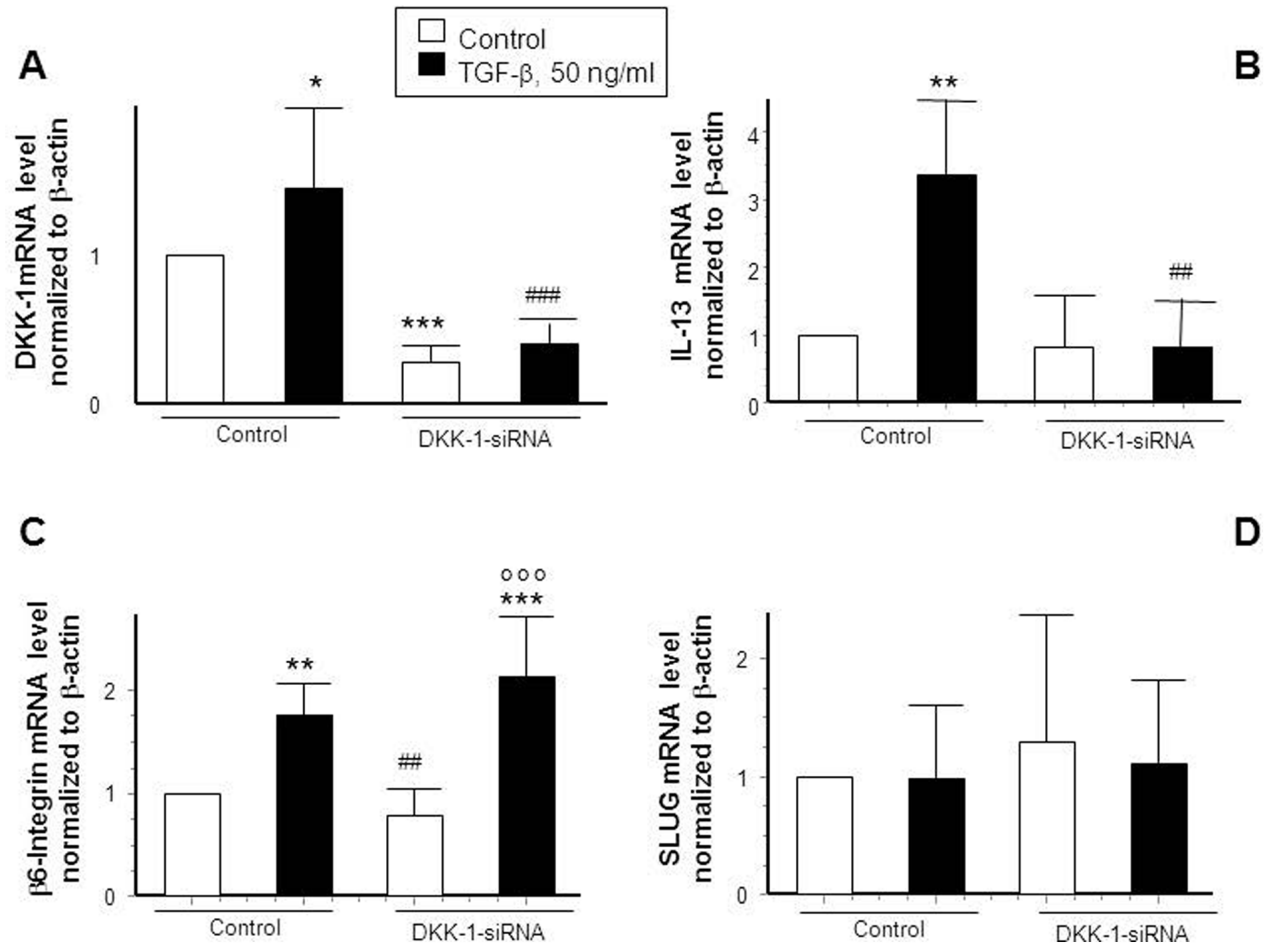
Knock-down of DKK-1 prevents TGF-β induced up-regulation of IL-13. (A) DKK-1 expression in control-transfected cells and after DKK-1-siRNA transfection with or without TGF-β (50 ng/ml) treatment (n = 5). (B) IL-13 (n = 4), (C) β6-Integrin (n = 5) and (D) SLUG mRNA expression (n = 4) in HT-29-IEC in control-transfected and transfected cells with or without application of TGF-β for 24 h. Asterisks indicate significant differences versus the respective control whereas # indicates significant differences versus TGF-β treatment and ° indicates significance in comparison to non-treated siRNA transfected cells. */# = p<0.05, **/## = p<0.01, ***/###/°°° = p<0.001.

We next studied the impact of DKK-1 on the expression of genes involved in fistula pathogenesis. Therefore, we transfected HT-29-IEC either with unspecific control siRNA constructs or with DKK-1 specific siRNA constructs and treated the cells with TGF-β (24 h, 50 ng/ml). As expected, presence of DKK-1-specific siRNA constructs clearly reduced DKK-1 basal mRNA levels and TGF-β was no longer able to induce DKK-1, implying a successful DKK-1 knock-down on mRNA level (Figure 3, A) as well as on protein level (Figure S2, B). Subsequently we assessed the expression pattern of IL-13, β6-integrin and the transcription factor SLUG in response to the growth factor (Figure 3, B–D). As expected, TGF-β induced IL-13 mRNA in DKK-1 competent cells. Of note, this effect was absent in DKK-1 knock-down cells (Figure 3, B). However, DKK-1 depletion had no significant effect on TGF-β induced up-regulation of β6-integrin (Figure 3, C) or on the expression of the transcription factor SLUG (Figure 3, D). These findings strongly suggest that TGF-β induces IL-13, but not β6-integrin expression in IEC via DKK-1.

### β-Catenin Regulates DKK-1 mRNA Expression in HT-29-IEC

**Figure 4:**
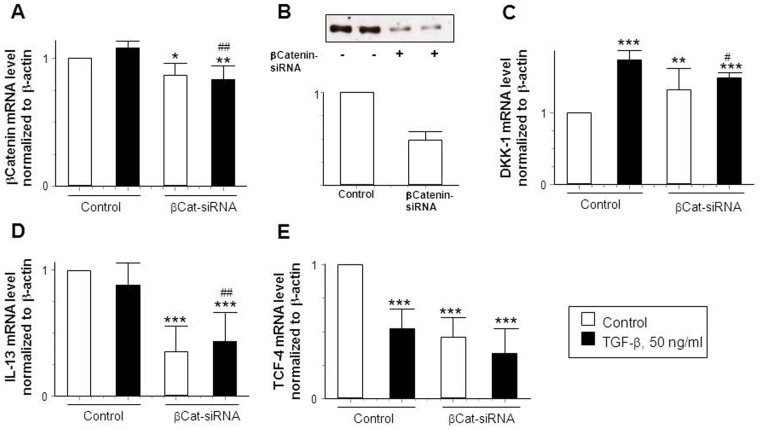
IL-13 levels are decreased in β-Catenin knock-down-cells compared to β-Catenin-competent cells. (A) mRNA expression and (B) protein levels of β-Catenin (n = 2), mRNA levels of (C) DKK-1, (D) IL-13 and (E)TCF-4 in control and β-Catenin-knock-down HT-29 IEC stimulated with or without TGF-β (50 ng/ml) for 24 h (n = 5 each experiment).Asterisks indicate significant difference versus the no stimulated control cells whereas # indicates significant differences versus TGF-β treated control cells. */# = p<0.05, **/## = p<0.01, *** = p<0.001.

Along with DKK-1, β-catenin plays a major role in the Wnt-signaling pathway. Hereby, β-catenin seems to be upstream of DKK-1 [28]. Therefore, we studied whether knock-down of β-catenin would alter TGF-β-induced increase in DKK-1 mRNA levels. We transfected HT-29-IEC with either non-specific or β-catenin-specific siRNA constructs. As shown in figure 4, β-catenin siRNA decreased β-catenin mRNA levels (Figure 4, A) as well as protein levels (Figure 4, B) significantly. Of note, the TGF-β induced increased in DKK-1 mRNA was ameliorated in β-catenin deficient cells (Figure 4, C). Surprisingly basal levels of DKK-1 mRNA were already elevated in untreated β-catenin deficient cells (Figure 4, C). Further, we found that untreated as well as TGF-β -treated β-catenin deficient cells express significantly less IL-13 mRNA compared to β-catenin competent IEC (Figure 4, D). Interestingly, TGF-β treatment decreased levels of TCF-4 transcription factor that interacts with β-catenin to regulate DKK-1 expression [35]. Further, we found a significant reduction of TCF-4 in untreated as well as TGF-β -treated cells lacking β-catenin (Figure 4, E), thus being consistent to the fact that nuclear complex formation of β-catenin/TCF-4 takes place during canonical Wnt-signaling pathway [28], [35]. These data strongly suggest that β-catenin regulates Dkk-1 mRNA expression in IEC in response to TGF-β and is, similar to Dkk-1, involved in the regulation of TGF-β-induced IL-13 expression in IEC. These observations could provide a functional role for β-catenin in the pathogenesis of CD-associated fistulae.

### IL-13 Minimizes DKK-1 Levels in Epithelial Cells and Fibroblasts

**Figure 5:**
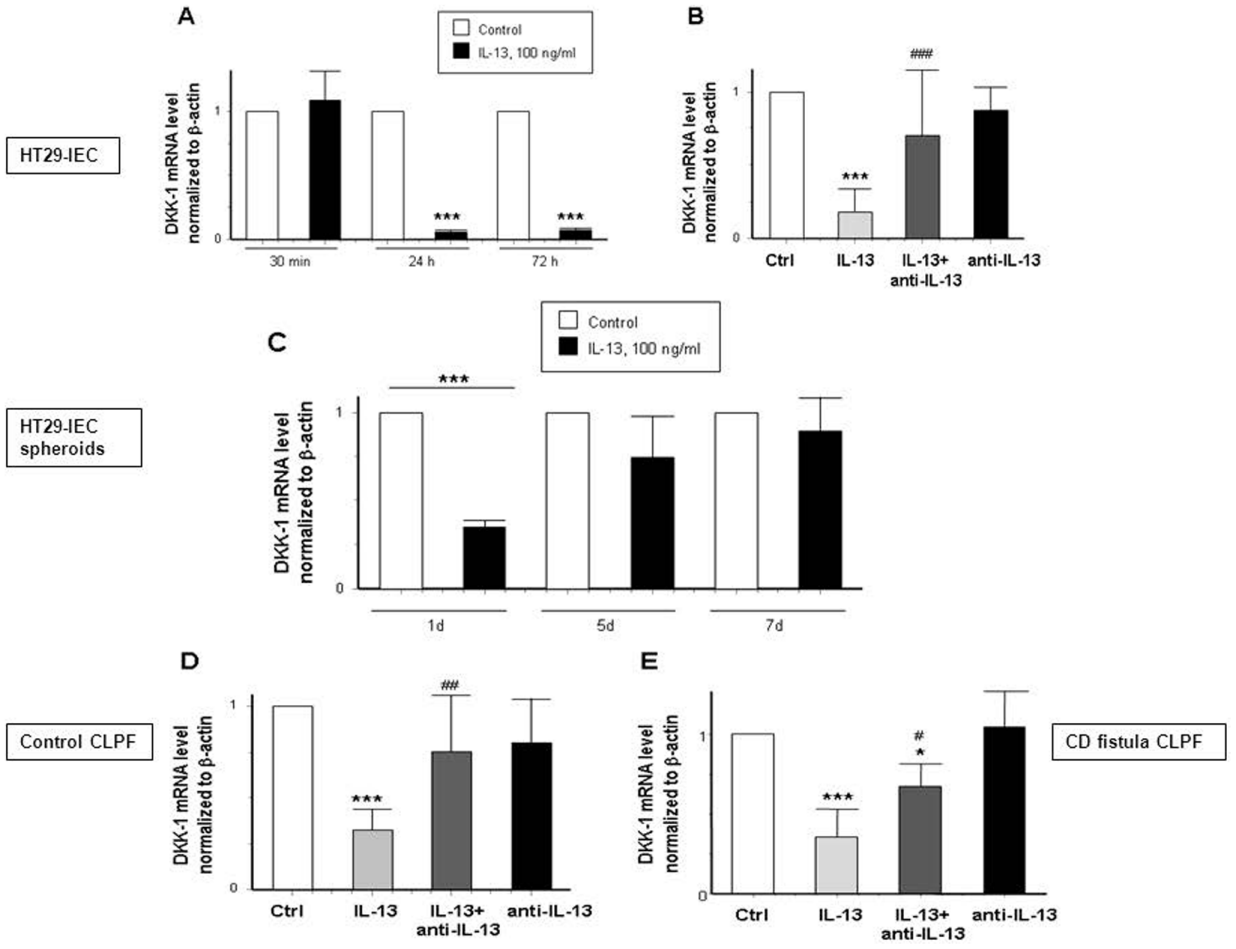
IL-13-induced decrease of DKK-1 mRNA expression is reversed by an anti-IL-13 antibody in IEC and in fibroblasts. (A) DKK-1 mRNA expression in HT-29 cells stimulated with IL-13 (100 ng/ml) for 30 min, 24 h and 72 h (n = 3). (B) Effect on DKK-1 levels in HT-29-IEC treated for 24 h alone with IL-13, an anti-IL-13 antibody (100 µg/ml) or a combination of both (n = 12). (C) Expression pattern of DKK-1 in HT-29-spheroids exposed to IL-13 for 1, 5 and 7 days (n = 3). (D) DKK-1 mRNA expression in intestinal control fibroblasts (n = 5) and (E) in CD- patients with a fistulizing course (n = 4). Asterisks indicate significant differences versus the respective control whereas # indicates significant differences versus IL-13 treatment. */# = p<0.05, ## = p<0.01, ***/### = p<0.001.

We have recently shown that synergisms between TGF-β and IL-13 might play a role in the pathogenesis of CD-associated fistulae [12]. Since we have shown that DKK-1 regulates TGF-β-induced expression of IL-13 in HT-29-IEC, we next investigated whether IL-13 might be able to regulate the expression of DKK-1 as part of a possible feedback mechanism. We treated HT-29-IEC, HT-29-spheroids and fibroblasts from either non-IBD control patients or patients with fistulizing CD with IL-13 (100 ng/ml). In a first step, we assessed different time points to evaluate the maximal alteration in gene expression (Figure 5, A). The cytokine lead to a significant decrease in DKK-1 mRNA expression levels after 24 and 72 h in HT-29 monolayers. As expected, this down-regulation could be reversed after 24 h by co-administration of an anti-IL-13 antibody (100 µg/ml) (Figure 5, B). In HT-29-spheroids, IL-13 decreased DKK-1 mRNA expression after 24 h and the IL-13-induced effect on DKK-1 was declining after 5 and 7 d (Figure 5, C). The effects of IL-13 on DKK-1 expression seen in HT-29-IEC and spheroids could also be detected in control and CD fistula fibroblasts (Figure 5, D–E). Here, IL-13 caused a down-regulation of DKK-1 mRNA in both groups. However, while anti-IL-13 treatment fully reversed this effect in control CLPF, the antibody had only a limited, but nevertheless significant, effect in fistula CLPF. The antibody alone had no effect on DKK-1 mRNA expression in all studied cell types. In summary, IL-13 treatment reduced levels of DKK-1 mRNA in IEC as well as in CLPF of healthy and fistulizing individuals. This effect could be reversed by an anti-IL-13 antibody implying a potential role of the cytokine in the onset and progression of fistulae.

### TNF-α and MDP Induce DKK-1 Expression in CD Fistula Fibroblasts

**Figure 6:**
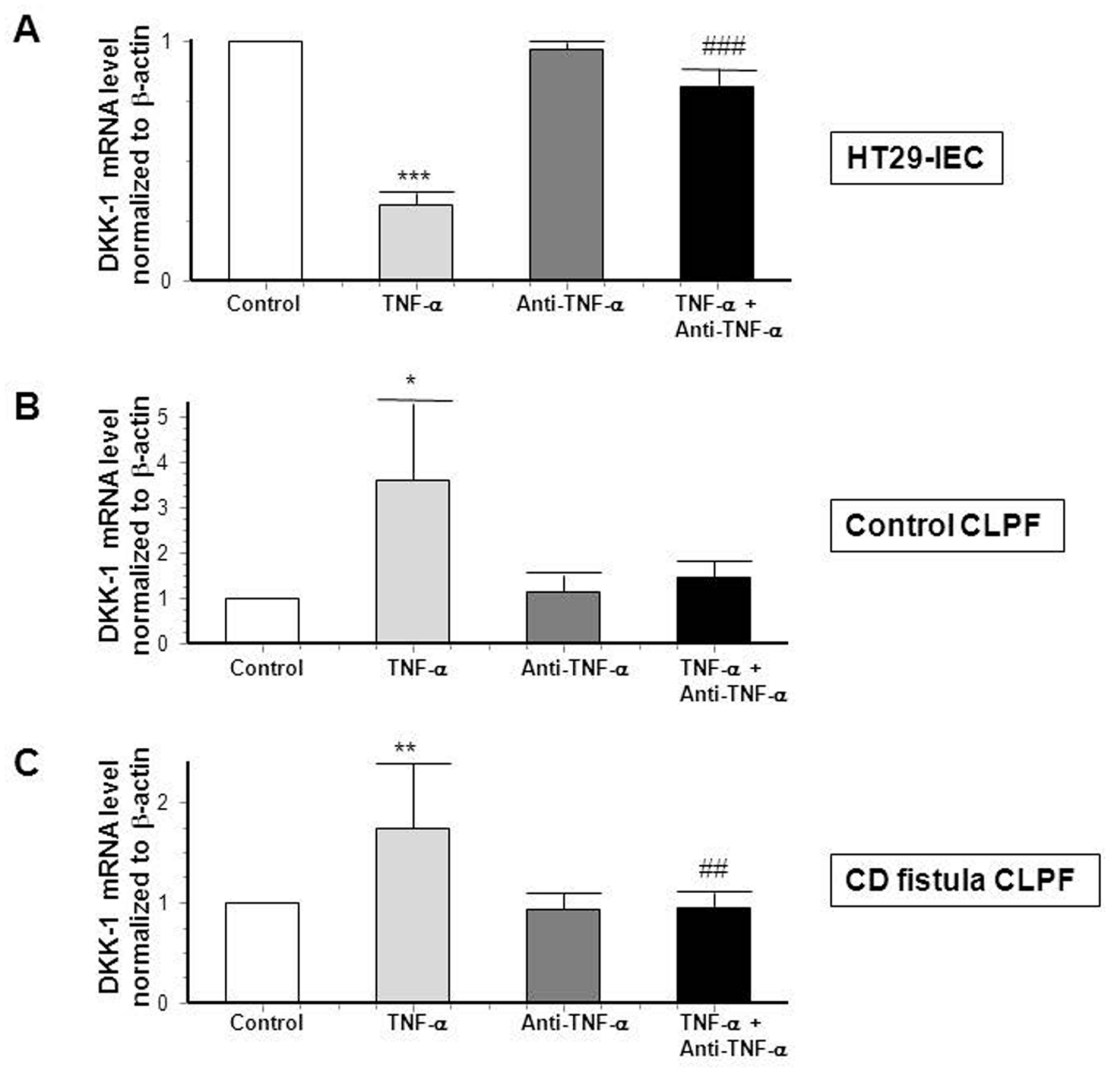
TNF-α inhibits DKK-1 mRNA expression in human IEC. (A) DKK-1 mRNA expression in response to TNF-α (50 ng/ml) and/or an anti-TNF-α antibody (24 µg/ml) for 24 h in HT-29-IEC (n = 3 for each experiment). (B) Control CLPF from healthy individuals (n = 3 for each experiment) and (C) CD CLPF (n = 4 for each experiment) in response to TNF-α (50 ng/ml) and/or an anti-TNF-α antibody (24 µg/ml) for 24 h. Asterisks indicate the difference versus the respective untreated control whereas # indicates significant differences versus TNF-α-treated cells. * = p<0.05, **/## = p<0.01, *** = p<0.001.

Since we have recently shown that TNF-α and TNF-receptor I are strongly expressed in and around CD-associated fistulae [11], we next investigated whether TNF-α could also affect DKK-1 mRNA expression in HT-29-IEC as well as in primary CLPF derived from healthy control subjects or CD-patients with a fistulizing disease course. In HT-29-IEC, administration of 50 ng/ml TNF-α for 24 h significantly reduced DKK-1 mRNA expression by more than 50%, (Figure 6, A). In contrast, treatment of CLPF of healthy control individuals and fistula fibroblasts with the cytokine significantly increased DKK-1 expression (Figure 6, B–C). These effects could be reversed when cells were co-treated with an anti-TNF-α antibody (Figure 6, A–C).

**Figure 7:**
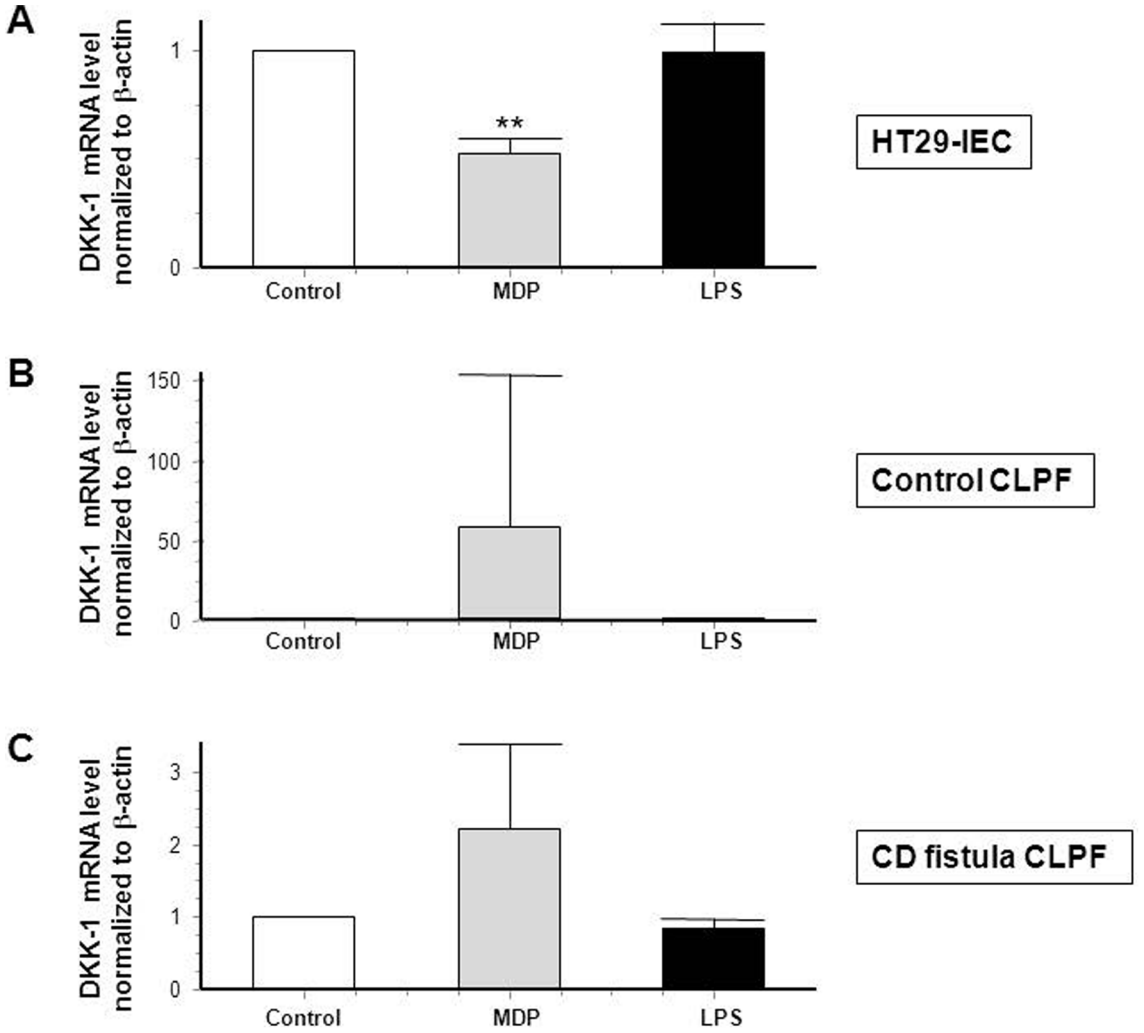
DKK-1 expression is decreased in IEC when MDP is present. DKK-1 mRNA expression in (A) HT-29-IEC (n = 3), (B) in control CLPF (n = 3) and (C) in CD CLPF (n = 4) in response to MDP (100 ng/ml) or LPS (100 ng/ml) for an exposition of 24 h. Asterisks indicate the difference versus the respective untreated control. ** = p<0.01.

Since numerous studies strongly suggest an involvement of bacterial components in CD pathogenesis and since the intracellular receptor for bacterial wall component muramyl dipeptide (MDP) [29], NOD2, is associated with a fistulizing disease course in CD [30], we further assessed the effects of MDP, but also the bacterial component lipopolysaccharide LPS on DKK-1 expression. Of note, MDP significantly decreased DKK-1 in HT-29 cells (Figure 7, A), but increased DKK-1 mRNA expression in healthy control fibroblasts and fistula fibroblasts of CD-patients (Figure 7, B–C) hereby resembling the effects of TNF-α. LPS had no effect on the expression of DKK-1 in all three cell types (Figure 7, A–C). Our data show that TNF-α as well as the bacterial wall component, MDP, down-regulate DKK-1 mRNA expression in IEC and TNF-α mediated effects can be neutralized by applying an anti-TNF-α antibody. In contrast, both stimuli increased DKK-1 mRNA levels in CLPFs of healthy control individuals and fistula fibroblasts of CD-patients. These findings suggest divergent regulatory mechanisms in IEC and CLPF and lead to the assumption that, besides TNF-α, bacterial components might also play a role in the pathogenesis of CD-associated fistulae.

## Discussion

In the present study we demonstrate increased DKK-1 levels in cells lining CD-associated perianal fistulae. Since DKK-1, as a part of the canonical Wnt/β-Catenin pathway, has already been shown to be involved in pathological processes such as cell migration, invasion and notably in EMT [24]–[26], [28], [31], this observation also supports the hypothesis that DKK-1 might be involved in EMT and consequent fistula formation in CD patients.

TGF-β exerted opposing effects on DKK-1 mRNA expression in IEC and CLPF. We show that DKK-1 is inducible by TGF-β in IEC, but it is down-regulated in response to the growth factor in fistula fibroblasts derived from CD-patients as well as in control fibroblasts derived from non-IBD control patients. This observation might be due to the fact that TGF-β induces EMT in IEC. However, fibroblasts are not capable of undergoing EMT and therefore, EMT-inducing effects of TGF-β might be altered or even reversed in these cell types. Of note, DKK-1 is a mediator of Wnt-signaling, hereby also mediating TGF-β-induced signaling events. Since TGF-β induces EMT in IEC also via Wnt-related pathways, it is high likely that DKK-1 represents an important regulator of TGF-β-induced EMT in IEC. In contrast, in fistula CLPF, EMT has already taken place. So, the activation of TGF-β-mediated signaling events that lead to the onset of EMT, are not necessary anymore and can be turned down, i.e. by the growth factor itself, resulting, among others, in the TGF-β-induced down-regulation of DKK-1. The molecular mechanism that IEC and CLPF feature different DKK-1 expression pattern in response to TGF-β might be due to epigenetic modifications within the DKK-1 gene locus.

A further hint to an important role for DKK-1 in the pathogenesis of CD-associated fistulae is the fact that DKK-1 knock-down prevents the TGF-β-induced up-regulation of IL-13 mRNA expression in IEC. We have previously shown that TGF-β induced IL-13 expression and secretion might play a crucial role in the pathogenesis of CD-associated fistulae [12]. Here, we found that TGF-β was no longer able to induce IL-13 expression in IEC when DKK-1 was knocked-down. This observation demonstrates that DKK-1 exhibits a regulatory function with respect to the TGF-β-mediated expression of IL-13. Further, when β-catenin was knocked-down, we also found a tendency showing that TGF-β-induced IL-13 expression is reduced. These findings are supported by *Notani et al.* demonstrating that β-catenin is required to induce IL-13 expression [32], but also by Koch et al. who found up-regulated β-catenin levels when epithelial cells are damaged [27], in example during chronic inflammation as observed during CD. All in all, our data support the hypothesis that TGF-β-mediated IL-13 stimulation is dependent on β-catenin and DKK-1 in IEC, demonstrating a functional role for both molecules in the pathogenesis of CD-associated fistulae.

Epithelial cells undergo EMT after being exposed to TGF-β, simultaneously inducing DKK-1 expression. On a functional level, elevated Dkk-1 levels in epithelial cells might aim for the inhibition of Wnt-signaling, meaning limitation of cell proliferation, migration and invasion. However, as we have shown, DKK-1 seems also to be required for TGF-β-induced IL-13 expression. These findings support on one hand the hypothesis that DKK-1 acts as an antagonist of TGF-β/Wnt-signaling. On the other hand, DKK-1 seems to be involved in mediating cell invasiveness by mediating the induction of IL-13 expression. Interestingly, IL-13 is also up-regulated by TGF-β during EMT and IL-13 actively suppresses DKK-1 expression. This means that TGF-β induces DKK-1 as an inhibitor of its own signaling cascades, namely Wnt-related signaling, but also induces IL-13 via DKK-1. Of note, IL-13 finally inhibits DKK-1 expression. According to our hypothesis, in a pathological setting featuring ongoing tissue damage such as chronic intestinal inflammation, the overwhelming secretion of TGF-β and the subsequent activation of signaling pathways associated with cell proliferation, migration and invasion might finally result in overwhelming secretion of IL-13. IL-13 then inhibits DKK-1 expression, what, in the end, contributes essentially to the establishment of EMT. As we have previously shown, IL-13 then induces genes associated with cell invasion, such as β6-integrin, thus finally leading to the onset of fistula formation. Further, fistula fibroblasts that already underwent EMT, feature a down-regulation of DKK-1 in the presence of TGF-β, further promoting Wnt-related signaling in these cells, what might finally lead to an advanced fistula formation.

In our experiments, we also detected decreased levels of DKK-1 in IEC after exposure to TNF-α. Of note, the cytokine had an opponent effect in perianal fistula fibroblasts derived from CD-patients. This result is consistent with data shown by Diarra et al., suggesting that DKK-1 can be induced by TNF-α in arthritis [33]. Of note, TNF-α can induce EMT in IEC [23] and has also been well documented to induce secretion of TGF-β [22]. Therefore, it seems plausible that the TNF-α-induced decrease in DKK-1 levels plays an essential role for mediating TNF-α-induced EMT, since down-regulation of DKK-1 obviously promotes the expression of genes being associated with cell proliferation and migration.

As already mentioned the inflammatory mediator IL-13 blocked DKK-1 expression not only in HT-29-IEC, but also in fibroblasts of healthy non-IBD subjects as well as CD associated fistula patients. This effect could efficiently be reversed in all investigated cell types by an anti-IL-13 antibody. These findings support our hypothesis that IL-13 could be an interesting target for fistula therapy. Notani et al. recently demonstrated that IL-13 expression is up-regulated via β-catenin [32]. We fully confirm this observation, since in our β-catenin knock-down experiments, we observed that IL-13 levels were constantly lower concomitant with TCF-4 in cells lacking β-catenin compared to β-catenin competent cells. This finding, in addition to the dual role of β-catenin as an transcription factor, but also as a part of the intercellular adherens junctions at the plasma membrane categorizes β-catenin as an interesting target to investigate in the pathogenesis of fistulae, where epithelial cells loose intercellular connections [34]. During EMT, cell-cell contacts are lost mainly seen by a loss of E-cadherin, but also by lower expression of cytoplasmic or membrane-bound β-catenin. During EMT, β-catenin instead translocates into the nucleus in response to TGF-β and forms a complex with TCF-4 in attempt to regulate target gene transcription [31]. The reduced β-catenin mRNA levels in IL-13-treated HT-29-IEC might be due to a negative feedback loop since β-catenin has already accumulated in the nucleus.

To study EMT in a model, that better resembles the in-vivo conditions, we analyzed the effects of indicated cytokines not only in monolayer cells but in three-dimensional multicellular structures, the aforementioned spheroids. This model is suitable for studying proliferation, differentiation, migration or cell death because of the high polarity of epithelial cells and the expression of junctional proteins [36]. A well-defined geometric structure enables us to macroscopically assess the spheroids and they provide us a direct correlation between structure, protein and gene expression [36] which is not possible in studying monolayer cells.

**Figure 8:**
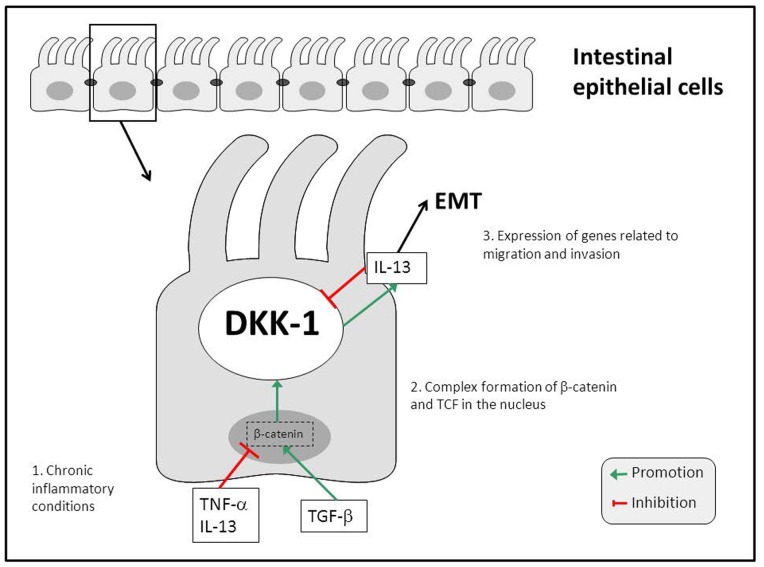
DKK-1 regulates TGF-β induced IL-13 secretion in CD-associated perianal fistulae. Under chronic inflammatory conditions, TNF-α, and also IL-13, are present at high levels and exert inhibitory effects on DKK-1 mRNA expression in IEC. TNF-α further stimulates TGF-β secretion that promotes DKK-1 mRNA expression in IEC. The effects of the cytokine and the growth factor on DKK-1 are mediated via β-catenin/TCF. On a functional level, TGF-β induces IL-13 secretion via a β-catenin/DKK-1-dependent pathway. These mechanisms through synergism between TGF-β and IL-13 finally lead to an enhanced expression of genes associated with EMT and therefore to the formation of perianal fistulae associated to CD.

In summary, we demonstrate that DKK-1 is involved in regulating processes that are crucially involved in the onset of EMT in IEC. By regulating DKK-1 expression, TNF-α, TGF-β and IL-13 appear to be involved in regulatory feedback loops controlling cell migration and invasion in the pathogenesis of CD-associated perianal fistulae via DKK-1 (Figure 8). We have further demonstrated that IL-13 secretion is to be regulated via DKK-1 in response to TGF-β. The observations that mRNA levels of DKK-1 are altered in response to the mediators TGF-β, TNF-α and IL-13, all of them being essentially involved in EMT-associated fistula formation, suggest that DKK-1 might also to be an interesting target in fistula therapy. All in all, our data provide strong evidence for an involvement of DKK-1 in the pathogenesis of CD-associated perianal fistulae.

## Supporting Information

Figure S1 Control staining against Dkk-1. (A) Positive control staining against Dkk-1 (black arrow) in human uterus tissue. (C) Negative control staining using just the secondary antibody in perianal fistula tissue. (B, D) Representative sections of (A, C). The white arrow indicates Dkk-1 negative areas. (A, C) Pictures were taken with a 50-fold magnification.
(TIF)

Figure S2 Western blotting analysis of Dkk-1 in control or DKK-1-siRNA-transfected HT-29 cells. (A) Dkk-1 protein expression of control and TGF-β-stimulated HT-29 cells for 24 hours (50 ng/ml). (B) Dkk-1 protein expression of control- and DKK-1-siRNA-transfected HT-29 cells.
(TIF)
