# Supplementary figures and images for: The Role for Dickkopf-Homolog-1 in the Pathogenesis of Crohn’s Disease-Associated Fistulae
Source: PLoS One. 2013 Nov 8;8(11):e78882. doi: 10.1371/journal.pone.0078882 (PMC3826763; doi:10.1371/journal.pone.0078882)

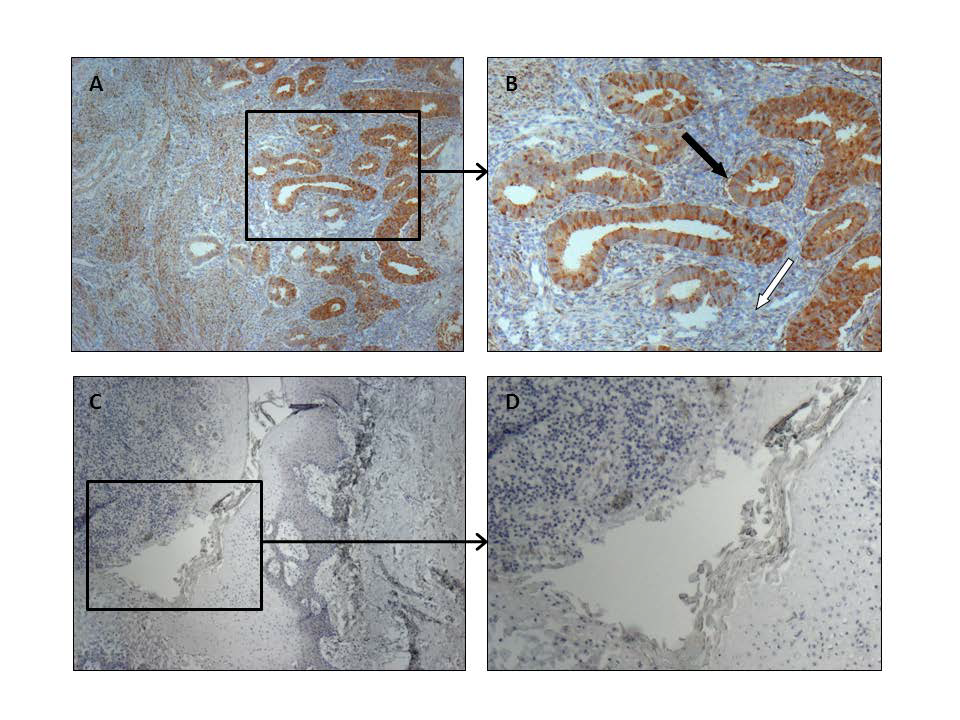

Supplement: Figure S1 — Control staining against Dkk-1. (A) Positive control staining against Dkk-1 (black arrow) in human uterus tissue. (C) Negative control staining using just the secondary antibody in perianal fistula tissue. (B, D) Representative sections of (A, C). The white arrow indicates Dkk-1 negative areas. (A, C) Pictures were taken with a 50-fold magnification. (TIF) [file pone.0078882.s001.tif]

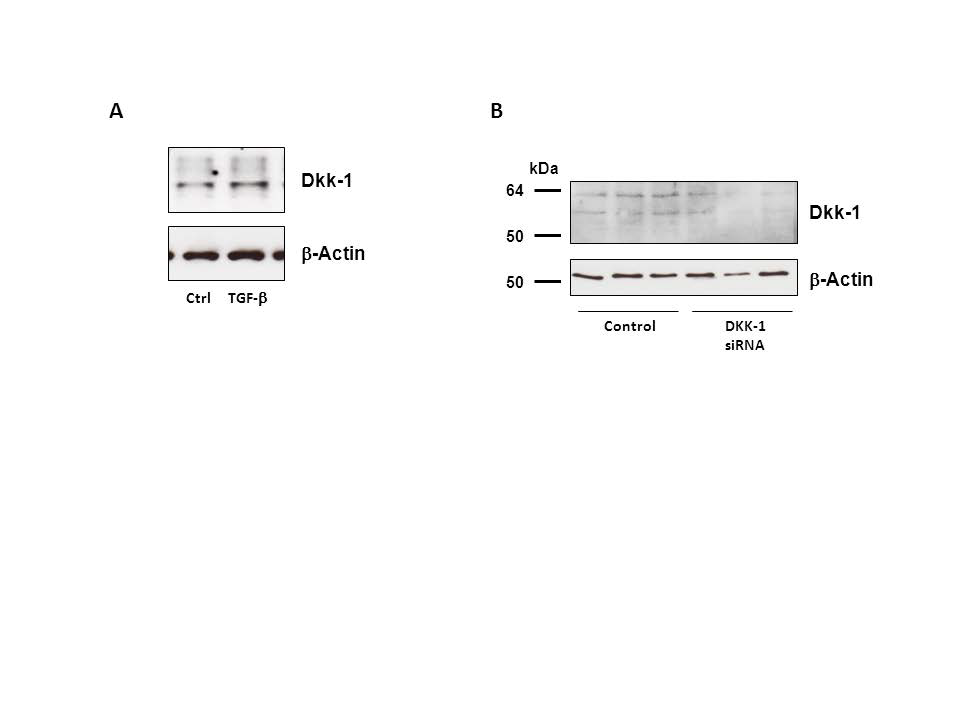

Supplement: Figure S2 — Western blotting analysis of Dkk-1 in control or DKK-1-siRNA-transfected HT-29 cells. (A) Dkk-1 protein expression of control and TGF-β-stimulated HT-29 cells for 24 hours (50 ng/ml). (B) Dkk-1 protein expression of control- and DKK-1-siRNA-transfected HT-29 cells. (TIF) [file pone.0078882.s002.tif]
